# Computational Identification of Gene Networks as a Biomarker of Neuroblastoma Risk

**DOI:** 10.3390/cancers12082086

**Published:** 2020-07-28

**Authors:** Lidan Sun, Libo Jiang, Christa N. Grant, Hong-Gang Wang, Claudia Gragnoli, Zhenqiu Liu, Rongling Wu

**Affiliations:** 1 Beijing Advanced Innovation Center for Tree Breeding by Molecular Design, Beijing Forestry University, Beijing 100083, China; sunlidan@bjfu.edu.cn (L.S.); jianglibo0534@163.com (L.J.); 2 Department of Public Health Sciences, Penn State College of Medicine, Hershey, PA 17033, USA; claudia.gragnoli@gmail.com; 3 Center for Computational Biology, College of Biological Sciences and Technology, Beijing Forestry University, Beijing 100083, China; 4 Division of Pediatric Surgery, Department of Surgery, Penn State College of Medicine, Hershey, PA 17033, USA; cgrant4@pennstatehealth.psu.edu; 5 Division of Pediatric Hematology and Oncology, Department of Pediatrics, Penn State College of Medicine, Hershey, PA 17022, USA; hwang3@pennstatehealth.psu.edu; 6 Division of Endocrinology, Diabetes, and Metabolic Disease, Translational Medicine, Department of Medicine, Sidney Kimmel Medical College, Thomas Jefferson University, Philadelphia, PA 19144, USA; 7 Molecular Biology Laboratory, Bios Biotech Multi Diagnostic Health Center, 00197 Rome, Italy

**Keywords:** gene regulatory network, neuroblastoma, idopNetwork, hub, gene co-regulation

## Abstract

Neuroblastoma is a common cancer in children, affected by a number of genes that interact with each other through intricate but coordinated networks. Traditional approaches can only reconstruct a single regulatory network that is topologically not informative enough to explain the complexity of neuroblastoma risk. We implemented and modified an advanced model for recovering informative, omnidirectional, dynamic, and personalized networks (idopNetworks) from static gene expression data for neuroblastoma risk. We analyzed 3439 immune genes of neuroblastoma for 217 high-risk patients and 30 low-risk patients by which to reconstruct large patient-specific idopNetworks. By converting these networks into risk-specific representations, we found that the shift in patients from a low to high risk or from a high to low risk might be due to the reciprocal change of hub regulators. By altering the directions of regulation exerted by these hubs, it may be possible to reduce a high risk to a low risk. Results from a holistic, systems-oriented paradigm through idopNetworks can potentially enable oncologists to experimentally identify the biomarkers of neuroblastoma and other cancers.

## 1. Introduction

Neuroblastoma, one of the most common cancers in children, is highly complex in terms of its genetic, physiological and clinical heterogeneity. It is this complexity that makes it extremely challenging to diagnose when and how neuroblastoma develops and further design the precise intervention [1,2]. In practice, several clinical parameters, such as age at diagnosis, tumor stage, genomic amplification of *MYCN* oncogene, and ploidy, are widely used as the markers of neuroblastoma risk [3,4,5,6]. Increasing studies have attempted to stratify neuroblastoma risk based on the pattern of differential gene expression [1,4,5,7,8]. Sets of genes of prognostic importance have been identified to link with neuroblastoma; for example, Oberthuer et al. [4] established a 144-gene predictor for classifying the stratification of neuroblastoma patients, Formicola et al. [5] used 18 genes to predict the outcome of stage 4 patients, and Utnes et al. [6] identified 20 mRNAs and six lncRNAs of clinical relevance to the prediction of tumor recurrence and response to neuroblastoma therapy.

Existing approaches for gene-based stratification are mostly based on the differential expression of single genes. As a widely used approach for general cancer research, such reductionist thinking can simplify the identification of key major genes for neuroblastoma risk [7]. It is becoming increasingly clear that a deeper understanding of cancer, including neuroblastoma, requires not only a detailed characterization of individual genes, but also of their interactions, which are encapsulated into an intricate but highly coordinated network [9,10]. As a complex disease, it is likely that neuroblastoma involves numerous genes that interact and work together to form a complex cellular network. As such, by regarding neuroblastoma as a network disease, we can develop and apply a holistic, systems-oriented approach to better reveal and interpret its genomic causes [11,12].

Computational tools play a critical role in reconstructing gene regulatory networks. Many approaches can only infer a single context-agnostic interaction network from a large number of samples, failing to reveal inter-sample heterogeneity [13,14]. Although several approaches have explored the possibility of reconstructing sample-specific networks [15,16], their application may be impaired by an incapacity to code a complete set of bidirectional, signed, and weighted interactions into a graph. Recovering such fully informative networks essentially requires the dynamic fitting of densely spaced temporal data [17], which are extremely difficult or even impossible to collect in practice; for example, multiple sampling and monitoring are not logistically permitted for cancer single-cell analysis and human gut microbiota studies. A majority of genomic studies conducted for cancer dissection in recent decades only have static data available.

More recently, Chen et al. [18] developed an interdisciplinary framework for reconstructing informative, dynamic, omnidirectional, and personalized networks (idopNetworks) using steady-state gene expression data from regular genomic experiments. The idopNetwork model can maximize the utility efficiency of genomic data to reconstruct biologically meaningful, context-specific, and large-scale but sparse networks. In clinical studies of graft remodeling, the use of idopNetworks has provided new insight into the genomic mechanisms of gene co-regulation that determine the success and failure of surgical operation. In this article, we apply and modify the idopNetwork model to reconstruct co-regulation networks of immune genes whose function may be related to neuroblastoma formation and identify which gene interactions determine high vs. low risk of this disease. As the proof of concept, idopNetworks characterize previously unknown gene interactions that can better serve as the biomarkers of neuroblastoma risk.

## 2. Results

### 2.1. Power Scaling Law and Functional Modularity

**Figure 1:**
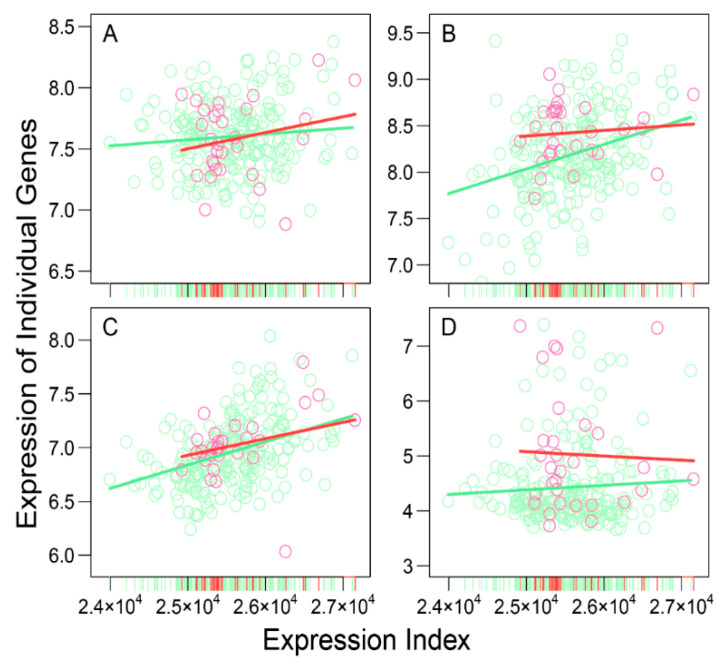
Allometric change in the expression level of four randomly chosen genes, *AATK* (**A**), *AKAP11* (**B**), *CD8A* (**C**), and *CDH9* (**D**), with expression index (EI) for low-risk (red) and high-risk patients (green). A dot denotes the observed expression value of a given gene in a sample, plotted over the EI of the sample, and a line represents the fitness of power equation to the EI-varying pattern of gene expression. The ticks at the *x*-axis indicate the EI of samples.

A total of 3439 immune-related genes were profiled for 247 neuroblastoma patients who differ in demographical and clinical factors, including age, gender, MYCN status, ploidy, stage, and overall (OS) and event-free survival (EFS). These patients are classified into two groups, high risk (*n*_1_ = 217) and low risk (*n*_2_ = 30). Traditional approaches aim to identify those genes that are expressed differentially between these two groups and use them as biomarkers to predict neuroblastoma risk. We argue that cancer biomarkers may not only be interpreted as single genes, but also include the pattern of how each gene interacts with every other gene to form a complex but organized network. Chen et al.’s [18] idopNetworks are among the most advanced networks to omnidirectionally reveal the topological differences of gene interactions across spatiotemporal gradients. To reconstruct such idopNetworks for neuroblastoma risk, we coin the concept of expression index (EI), defined as the total expression amount of all genes on a patient or sample, and plot the expression of individual genes against the ordered EI across samples (Figure 1). We found that high-risk patients cover a long range of EI from 2.4 × 10^4^ to 2.7 × 10^4^, whereas low-risk patients reside in the upper two-third of the range, regardless of their demographical and clinical features. This difference suggests that the considerably low overall expression level of the immune genes can predict high neuroblastoma risk, but their high-level expression does not necessarily imply the low risk of this cancer. A more precise predictive model of neuroblastoma risk may require the implementation of gene networks.

**Figure 2:**
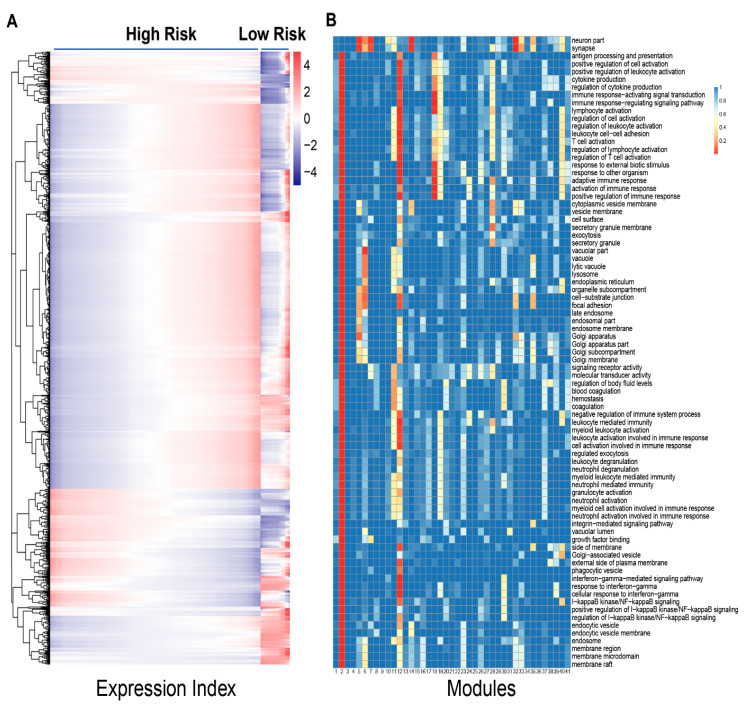
Functional clustering of 3439 genes into 41 distinct modules across 247 samples from high- (217) and low-risk groups (30) (**A**). Genes within each of the 41 modules are annotated for their biological functions (**B**).

Power equation is shown to broadly fit the trend of how individual genes change their expression levels over the EI. For example, as illustrated in Figure 1, four randomly chosen genes increase their expression levels gradually with the EI, but with different slopes of increase. Some genes, such as AATK and CD8A, display a similarity in EI-varying pattern between high- and low-risk groups (Figure 1A,C), but the others, e.g., AKAP11 and CDH9, are very group-specific (Figure 1B,D). We calculated the residuals of each gene and plotted them over the predicted values (Figure S1), whose independence suggests the statistical robustness of power fitness. We implemented Kim et al.’s [19] functional clustering to group all genes into 41 functional modules according to their similarity in EI-varying pattern (Figure 2A). Each module is composed of a different number of genes, and based on gene enrichment analysis, biological functions for the compositional genes are found to vary from module to module (Figure 2B). For example, module 2 contains genes that span a wide range of biological functions, including membrane raft, endocytic vesicle, neutrophil-mediated immunity, leukocyte-mediated immunity, and myeloid-leukocyte-mediated immunity, among others. Module 18 is composed of genes that specifically activate and regulate immune response through signal transduction and signaling pathway. Several modules, such as 3, 4, 9 and 36, include genes whose biological function is not identified. 

**Figure 3:**
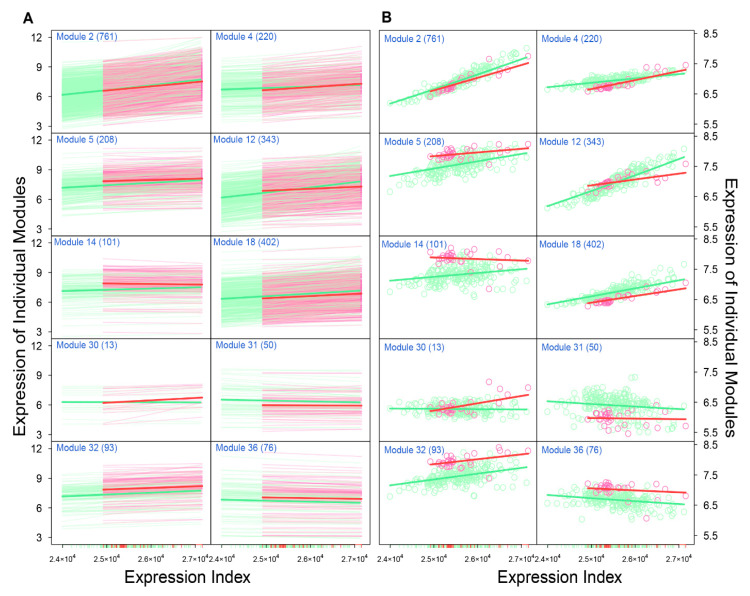
Distinct EI-varying pattern of ten chosen modules separately for high- (green) and low-risk groups (pink). (**A**) Power fitting of each gene within a module, shown as the change of gene expression across the EI. Each thin line represents a gene and thick lines stand for the mean fitting of all genes from the same modules. (**B**) Observed expression change of genes within a module across the EI (dot), fitted by the power equation (line).

We found that different modules display distinct EI-varying patterns of gene expression across samples. We show such discrepancies by choosing 10 modules as examples (Figure 3). Most modules, such as 2, 4, 5, 12, 18, and 32, increase their expression levels with the EI for both high- and low-risk groups, although the slope of increase depends on group type. Some modules, such as 31 and 36, slightly decrease their expression levels with the EI. In some modules, gene expression increases with the EI in one group but decreases in the other group (e.g., module 14 vs. 30). The pattern of how two groups differ in the amount of gene expression depends on the type of module. Some modules, like 2 and 4, have a similar amount of gene expression over the EI. The expression amounts of modules 5, 14, and 32 are clearly higher in the low-risk group than in the high-risk group. In these modules, genes are generally more strongly expressed in low-risk patients than in high-risk patients at a given EI. We also found a few modules, like 18 and 31, which display a higher expression level in high-risk than in low-risk patients. Overall, compared with the total expression level of all genes, the expression pattern of individual modules can more precisely serve as biomarkers of neuroblastoma risk.

### 2.2. Coarse-Grained Idopnetworks

In theory, all genes considered interact with each other to form a 3439-node regulatory network. However, because of the possible existence of network communities, we need to test whether there are distinct subnetworks within this large network. Genes within each subnetwork are linked more tightly and are therefore more similar to each other than those from different subnetworks. This allows us to detect subnetworks based on modularity analysis. Forty-one modules identified from functional clustering each represent a subnetwork and, thus, the 3439-node network is composed of 41 subnetworks or network communities. Different communities may be linked with each other through their compositional genes to form coarse-grained networks. Accordingly, subnetworks among genes within modules are called fine-grained networks.

**Figure 4:**
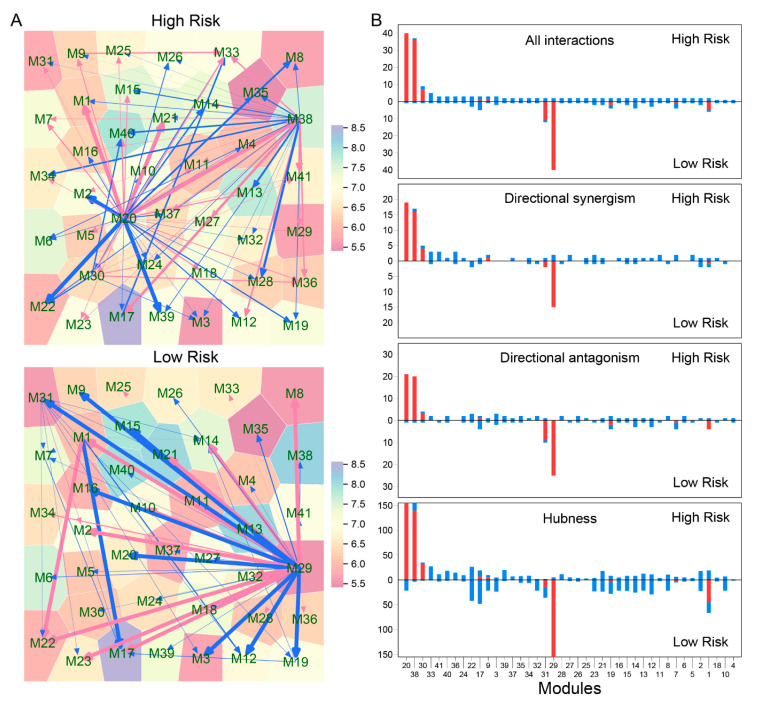
idopNetworks identified at the module level. (**A**) Networks among 41 modules for high- and low-risk groups. Arrowed red and blue lines denote promotion and inhibition, respectively, with the thickness of lines being proportional to the strength of promotion or inhibition. (**B**) The distribution of the total number of interactions, the number of directional synergism, the number of directional antagonism, and the hubness across modules for high- (upper) and low-risk groups (lower). Red and blue bars denote outgoing links (regulators) and incoming links (regulated genes), respectively.

We constructed coarse-grained idopNetworks at the module level for high- and low-risk groups, respectively (Figure 4). We found that such idopNetworks are strikingly different in topological architecture between two groups (Figure 4A). In the high-risk network, module 20 and 38 are hub regulators that are linked to many different regulated modules, but they become peripheral in the low-risk network, where modules 29 and 34 are hub regulators. From GO analysis, we did not identify known biological functions of genes involved in these hub modules (Figure 2B), suggesting that these genes as regulators promote or inhibit other genes that exert more direct impacts on a variety of biological processes. We found that the number of links for all 41 modules, except for 20, 38, 29 and 34, is broadly similar between the high- and low-risk networks (Figure 4B). This suggests that the difference in the qualitative structure of these two types of networks is determined by these hub modules. By testing the distribution of the total number of interactions using the power law, we found that the networks identified may be scale-free in both high- and low-risk groups. The pattern of interactions among modules is predominated by directional synergism and directional antagonism, which is consistent with widely identified cyclic synergism or antagonism (e.g., cyclic dominance) in nature [20].

In a regulatory network, hub genes are thought to play a central role in coordinating gene-gene interrelationships. We create the concept of quantitative hubness to describe the degree with which any specific gene can serve as a hub. As expected, the hub modules display remarkable differences in hubness between the high- and low-risk networks. However, it is interesting to find that the hubness of non-hub modules varies considerably from low-risk to high-risk groups. Taken together, the high-risk network distinguishes from the low-risk network in not only their qualitative structure, determined by a few mainstays, but also their quantitative organization, established jointly but differently by all components.

### 2.3. Dissection of Observed Expression Profiles

**Figure 5:**
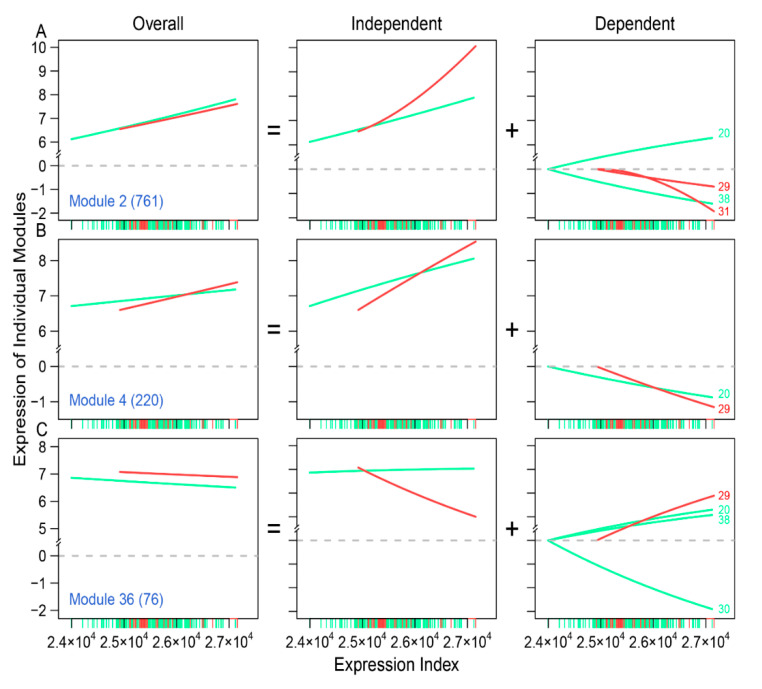
Decomposition of the overall expression level of three selected modules, 2 (**A**), 4 (**B**), and 36 (**C**), as regulated genes, into its independent and dependent components in low-risk (red) and high-risk patients (green). The dependent components of a regulated gene are determined by regulators with names given at the end of lines.

Traditional approaches can only compare the overall differentiation of gene expression between different regimes, but idopNetworks dissect the overall expression of any gene into its two underlying components: independent and dependent. By choosing three modules, we analyze these two components and show how each of them contributes to differentiated expression between high- and low-risk groups (Figure 5). Module 2 displays a similar overall amount and trend of EI-varying expression between two groups, but its independent component is expressed more increasingly with the EI in low-risk than high-risk groups (Figure 5A). For low-risk patients, the total dependent component of module 2 is negative because it is consistently inhibited by modules 29 and 31. For this reason, the observed expression level of module 2 in the low-risk group is lower than the degree with which this module can be expressed when it is isolated from modules 29 and 31. For high-risk patients, module 2 is inhibited by module 38 but also consistently promoted by another module 20, making its total dependent component a near-zero value. 

The independent component of module 4 has a sharper slope of EI-increasing gene expression curve in low-risk than high-risk group (Figure 5B). However, because this module is inhibited by other modules, to a greater extent in the low-risk than in the high-risk group, its overall expression amount, observed to be lower than its independent component, tends to be similar between the two groups. Genes in module 36 are observed to be more strongly expressed on low-risk than high-risk patients (Figure 5C), but their independent components vary dramatically between the two groups, with a sharp decrease curve for low-risk patients but a slight increase curve for the high-risk group. This difference is due to different patterns of interactions that are at play between these two groups. The expression of module 4 is promoted by module 29 in low-risk patients, and promoted by modules 20 and 38, but heavily inhibited by module 30. Taken together, if we predict neuroblastoma risk based on the expression level of module 36, as observed from its remarkable difference between low-risk and high-risk patients (Figure 5C), we should incorporate the impacts of modules 29, 20, 38, and 30 because these regulators contribute strikingly to low-risk vs. high-risk divergence in various ways. It should be noted that many of these regulators may also affect other modules, e.g., 2 (Figure 5A) and 4 (Figure 5B). If these regulated modules serve as regulators, we need to consider a complete network of interactions as a biomarker of neuroblastoma risk.

**Figure 6:**
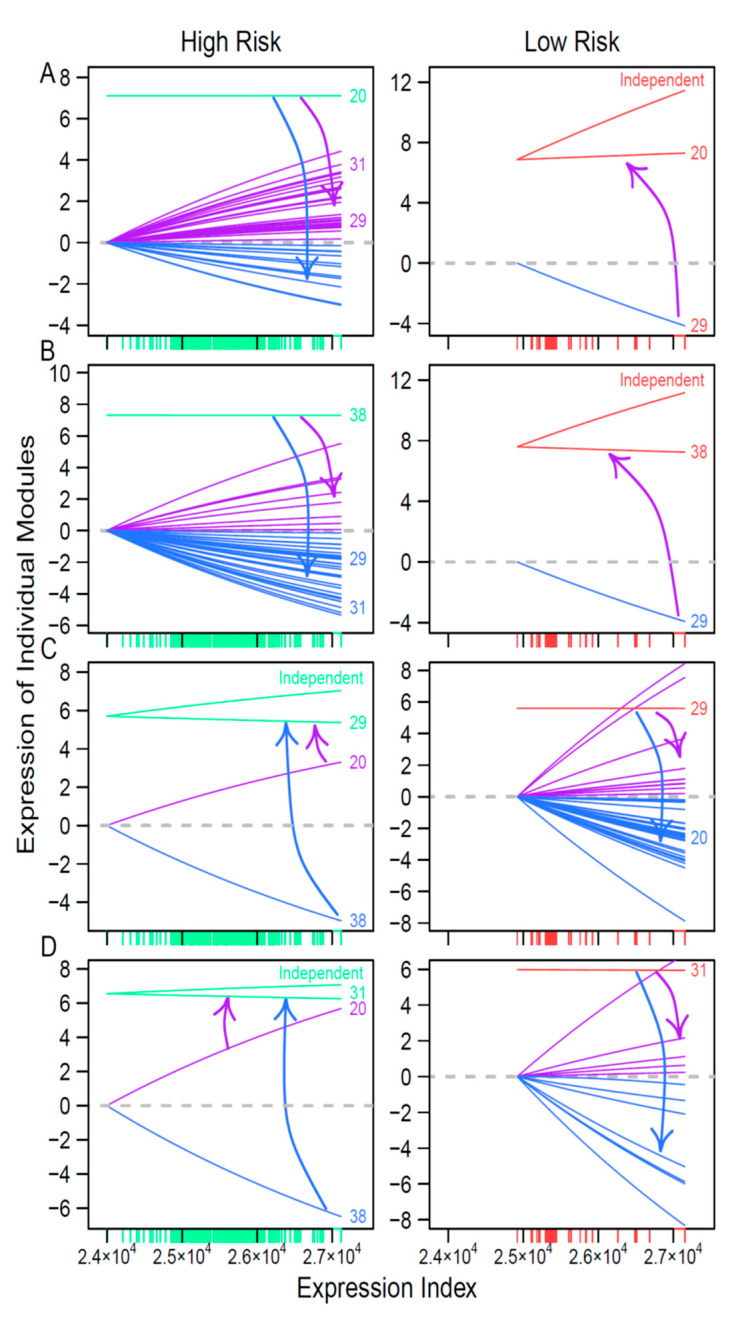
Outgoing and incoming regulation of hub regulators across the EI. Upper two panels: Modules 20 (**A**) and 38 (**B**) as hubs in the high-risk network regulate many other modules through positive promotion (purple lines) or negative inhibition (blue lines), but become regulated (inhibited, blue line) in the low-risk network. Lower two panels: Modules 29 (**C**) and 31 (**D**) as hubs in the low-risk network regulate many other modules through positive promotion (purple lines) or negative inhibition (blue lines), but become regulated (promoted, red line; inhibited, blue line) in the high-risk network. The direction at which one module regulates other module(s) is indicated by arrows. Green lines and red lines denote the expression change of hub modules in the high- and low-risk networks across the EI, respectively. For those regulated hub modules, the difference between their overall expression and independent expression is indicated.

To show how regulators affect other modules differently between the two risk groups, we draw the EI-varying curves of these regulators and their influences (Figure 6). In the high-risk network, modules 20 and 38 are two hub regulators (Figure 3), each of which promotes or inhibits the expression of many other modules, including modules 29 and 31, whereas, in the low-risk network, modules 20 and 38 are subordinates, regulated by module 29 (Figure 6A,B). Modules 29 and 31 are hub regulators in the low-risk network, but they are regulated by modules 20 and 38 (Figure 6C,D). Taken together, the reciprocal change in hubs as leaders may be a driver of differences between high- and low-risk networks. The hubs of one network are reciprocally regulated by their subordinates in the other network.

### 2.4. Fine-Grained IdopNetworks

**Figure 7:**
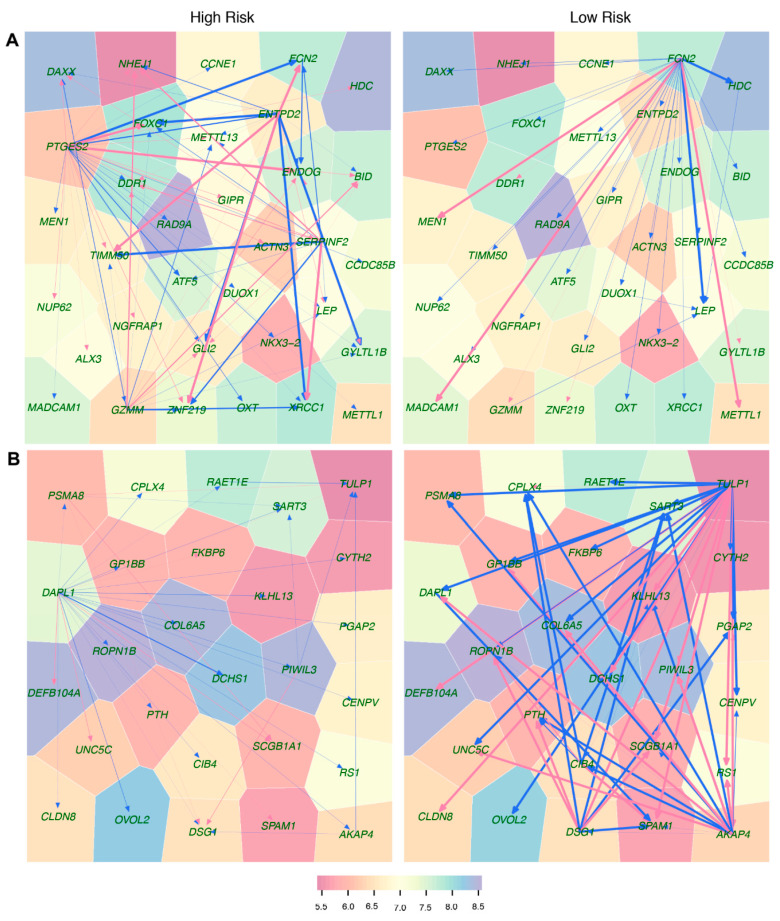
Voronoi treemaps that visualize fine-grained idopNetworks among genes from module 20 (**A**) and 29 (**B**) for high-risk and low-risk patients. Each polygon area (node) is represented by a gene (with its name shown), with the color metric being proportional to the overall expression level of this gene. Activation and inhibition are denoted by arrowed red and blue lines, respectively, with the thickness of lines being proportional to the strength of gene–gene interactions.

We reconstructed 41 fine-grained subnetworks each for a different module. The qualitative difference of coarse-grained networks between two types of patients includes the reciprocal change in the leadership of modules 20 and 29 and their regulatory relationship. We choose these modules to characterize and interpret how fine-grained idopNetworks vary between low-risk and high-risk groups (Figure 7). Genes interact with each other in ways that are extremely different between two types of patients. The network of module 20 is structurally much simpler for low-risk than high-risk patients; the low-risk network contains only one regulator *FCN2* that plays a dominant role in promoting or inhibiting all genes, whereas the high-risk network is constituted by multiple regulators, such as *PTGES2*, *ENTPD2*, *SERPINF2*, and *GZMM*, and many followers, such as *TIMM50*, *GLI2*, *ZNF219*, *ENDOG*, and *XRCC1*, which are affected simultaneously by several regulators (Figure 7A). It is likely that this difference leads the high-risk network to be more stable than the low-risk network for module 20, which thus explains why this module serves as a leader in the high-risk coarse-grained network but a subordinate in the low-risk coarse-grained network (Figure 6A). Being a regulator of the lectin complement pathway, *FCN2* contributes to innate immune response and is expressed at low levels in ovarian tumors compared to normal ovaries [21]. However, for high-risk patients, FCN2 loses its readership, and is heavily regulated by *PTGES2* and *ENTPD2*. In the meantime, *PTGES2* activates the ENDOG gene implicated in cancer, aging, and neurodegenerative diseases [22] and *ENTPD2* activates the ZNF219 gene as a member of the Sox9-assembled transcriptional factory participating in chondrocyte differentiation [23]. 

Risk-specific difference in the structure of fine-grained subnetwork for module 29 supports the postulation above, i.e., this module as a leader of low-risk coarse-grained network (Figure 6B) involves multiple regulators and multiple followers, whereas it, as a follower in the high-risk network, is dominated only by a single regulator (Figure 7B). *DARL1* is a dominant regulator of the high-risk subnetwork, widely inhibiting the expression of many immune genes. This regulator encodes the first member of the ARF-like protein subfamily in the secretory pathway, found to be lethal if it is deleted in *Drosophila* [24]. In the low-risk subnetwork, *DARL1* is inhibited and activated by two major regulators, *TULP1* and *AKAP4*, respectively, thus its overall expression is determined by the relative strength of inhibition and activation. Both *SART3* and *CPLX4* are jointly inhibited by *AKAP4*, *DSG4*, and *CIB4*. *SART3* is a gene encoding an RNA-binding nuclear protein that is a tumor-rejection antigen containing tumor epitopes [25]. Because of its capability to induce HLA-A24-restricted and tumor-specific cytotoxic T lymphocytes in cancer patients, this antigen is used for specific immunotherapy. The protein encoded by *CPLX4* may be involved in synaptic vesicle exocytosis [26]. Taken together, complex relationships among genes from module 29 may be related to low neuroblastoma risk.

Some modules contain many genes, e.g., there are as many as 761 genes in module 2. Thanks to its statistical power, the idopNetwork model can still reconstruct a large-scale subnetwork for these modules. Figure S2 illustrates 761-node subnetworks of module 2 for low-risk and high-risk patients, where risk-specific differences in the structure and organization of gene interactions can be identified. Figures S3 and S4 show the subnetworks of modules 38 and 31, respectively, for patients at low- and high-risk levels.

## 3. Discussion

Differentiated expression of genes has been widely used as an approach for stratifying neuroblastoma risk. Although single gene analysis has proven its power for risk stratification, we argue that neuroblastoma risk includes multiple genes that interact with each other through intricate but coordinated interaction networks. Traditional approaches for inferring informative networks, i.e., those coded by bidirectional, signed, and weighted interactions, rely on high-density temporal data which are hardly available in most cancer studies. These approaches can also only reconstruct context-agnostic networks, failing to reveal the change in network structure in response to environmental and developmental signals. We implement and modify Chen et al.’s [18] networking model to recover informative, dynamic, omnidirectional, and personalized networks (idopNetworks) from static expression data and compare how idopNetworks vary structurally and organizationally over biological and environmental regimes. By incorporating functional clustering and variable selection, idopNetworks can be identified at any dimension and scale and with any topological complexity. All these advantages are especially essential and of the utmost importance for neuroblastoma-gene networking because this disease involves multitudinous genes interacting in a complex, dynamic manner.

As the proof of concept, we analyzed publicly available data containing 3439 genes expressed in neuroblastoma patients to show the potential of idopNetworks as a predictive biomarker of genomic differences between high- and low-risk patients. In reconstructing idopNetworks, we found that neuroblastoma risk can be predicted at three levels. First, the premise of reconstructing idopNetworks is to define the concept of expression index defined as the total amount of gene expression in a sample. We found that the expression index of low-risk patients spans the upper two-third of high-risk patients (Figure 1), showing the possibility to assess those in the lower one third of expression index as high-risk patients. Second, among a total of 41 distinct gene modules classified by functional clustering, we found that some modules change their expression pattern over expression index differently between two groups of patients (Figure 2). As thus, these modules can be potentially used to predict neuroblastoma risk.

Third, relative to the two biomarkers above, idopNetworks provide a more precise predictor of neuroblastoma risk. We dissect a 3439-node network into 41 subnetworks based on module classification. The network of these subnetworks reflects how gene modules interact with each other to form a coarse-grained network, while each subnetwork characterizes interactions among individual genes within a fine-grained network. We found that directional synergism and directional antagonism overwhelmingly dominate both coarse- and fine-grained networks. Evolutionary studies suggest that commensalism (analogous to directional synergism) and amensalism (analogous to directional antagonism) are the two most economic strategies for animals to interact with others in nature [20]. We speculate that this phenomenon may also occur for genes to choose their interaction strategies in a regulatory network.

We found that both coarse- and fine-grained networks show dramatic structural and organization differences between low- and high-risk patients. We characterized the role of key hub regulators in driving how high-risk patients differ from low-risk patients. These regulators promote or inhibit the expression of numerous genes that are directly involved in immunity processes towards neuroblastoma pathogenesis. We identified different hubs that mediate low- and high-risk networks. For example, modules 29 and 31 are hubs in the low-risk network, whereas hubs in the high-risk network become modules 20 and 38. It is interesting to find that hubs in one coarse-grained network are reciprocally regulated by those in the other coarse-grained network, showing that the leadership change may be an important driver of neuroblastoma risk. The detailed biological functions of these hubs deserve further investigation.

Through idopNetworks, we can determine how gene–gene interactions can be used to serve as a predictor of neuroblastoma risk. For example, module 2 shows no difference in the dynamic pattern of gene expression between two groups of patients (Figure 5A), suggesting that this module cannot serve as a biomarker. Yet, by decomposing the overall expression of this module into its independent and dependent components, we found that the independent component increases its expression with expression index much more strikingly for low-risk than for high-risk patients. Thus, the dynamic pattern of the independent component can be potentially used to predict neuroblastoma risk. In practical clinics, neuroblastoma risk can be reduced by designing target interventions to obstruct the expression of modules 29 and 31 that inhibit module 2. Similarly, the independent component of module 36 is associated with reduced neuroblastoma risk, which can be strengthened by artificially inhibiting the expression of module 29 that activates module 36. Taken together, the multiplayer game model derived from idopNetworks may provide a more precise predictive means of neuroblastoma risk and solid scientific guidance on drug design for neuroblastoma control.

We reconstructed neuroblastoma-related idopNetworks based on gene expression data. These networks may serve as a starting point to explore the biological and functional relevance of key gene-gene interactions for neuroblastomas. Although GO analysis shows the biological relevance of some gene interactions detected by the model, close collaboration with experimental oncologists is crucial for not only further justifying the model but also making novel discoveries. In addition, mounting evidence indicates that, in addition to transcriptional dysregulation, cancer may also be predicted by epigenetic, protein, or metabolite biomarkers [27,28]. By incorporating these omics data, idopNetworks can be extended to infer multiscale and hierarchic networks, which can better interrogate the biological mechanisms that drive neuroblastoma. This work focuses on the comparison of idopNetworks between different neuroblastoma risks, but it should be expanded to compare network differences determined by other biological or environmental regimes, such as death vs. survival. In idopNetwork inference, we combine patients from the same group, thus neglecting genetic differences among patients. As cancer cells hosted on patients, neuroblastoma may not only be controlled by host genetics, but also somatic mutations of tumor cells. While many studies have identified a number of host loci for cancer based on GWAS, Sang et al. [29] developed a micro-GWAS model to detect mutation loci that affect tumor phenotypes. The integration of idopNetwork and Sang et al.’s [29] micro-GWAS will shed light on why and how neuroblastoma is formed, knowledge that can greatly facilitate the design of personalized neuroblastoma therapies.

## 4. Methods

### 4.1. Design of Genomic Experiment for Neuroblastoma Risk

Consider a set of *n* sampled neuroblastoma patients, sorted into two prognostic subgroups with expected high and low risk of death from disease. These samples are stratified according to demographical and clinical factors, such as age, gender, MYCN status, ploidy, stage, and overall (OS) and event-free survival (OS). Suppose a total of *m* genes are profiled and measured for their expression amounts on all samples. Let $g_{ij}$ denote the expression level of gene *j* on sample *i* (*i* = 1, …, *n*). We consider each sample as an ecosystem composed of *m* interacting genes. Thus, we define the total expression of all genes on a sample, i.e., $E_{i}=\sum_{j=1}^{m}g_{ij}$, as its expression index (EI) that reflects the sample’s capacity to carry essential resources for the simultaneous expression of all genes. Based on the definition, $g_{ij}$ and $E_{i}$ establish a part-whole relationship across samples and, thus, $g_{ij}$ can be understood as a function of $E_{i}$, which is equivalently denoted as $g_{j}\left(E_{i}\right)$. This part-whole relationship theory forms an analytical basis for reconstructing regulatory networks for high- vs. low-risk neuroblastoma.

### 4.2. Modularity Detection by Power Law-Based Functional Clustering

Modular organization is a generic property of gene networks, recognized as a design principle of biological systems. A module or community is a subset of nodes that are more densely connected with each other than with those nodes from the remaining network [30]. Modules, as function units, are pervasive in a range of biological processes, such as cell differentiation, metabolism, cell cycle, and signal transduction. Evidence shows that modularity structure enhances the adaptability and robustness of biological systems to perturbations. The existence of gene modules alerts us not to attempt to link each pair of genes in the network, but to detect gene subnetworks or communities. The modularity theory avoids computational prohibition when the number of genes considered is very large.

We develop a top-down approach for modularity detection by breaking down a whole network into its compositional subnetworks in a reverse engineering fashion. This approach is based on the functional clustering of all genes into different modules according to their EI-varying functional similarity. As stated above, $g_{j}\left(E_{i}\right)$ is part of $E_{i}$, whose relationship is thought to obey the power law, as universally observed in nature [31,32,33]. Thus, how $g_{j}\left(E_{i}\right)$ scales with $E_{i}$ across samples is mathematically described as

(1) $$g_{j}\left(E_{i}\right)=\alpha_{j}E_{i}^{\beta_{j}}$$

where $\alpha_{j}$ and $\beta_{j}$ are the constant and allometric exponent for gene j that changes its expression level with EI. We incorporate Equation (1) into a mixture functional clustering algorithm [19] by which we cluster all m gene into different modules. The EI-varying pattern of gene expression is more similar within than between modules, leading genes within modules to be densely linked but those between modules to be sparsely linked. We implement an information criterion, such as AIC or BIC, to determine the optimal number of modules (denoted as *L*) for a given set of genes.

With the information about gene modules, we will reconstruct coarse-grained regulatory networks among genes from between modules, and fine-grained regulatory networks among genes within modules. We cluster genes based on how they are expressed differently across a full range of samples from both risk groups. It is possible that these two groups change their expression across samples in a different manner. Thus, we need to reconstruct different types of networks each for a risk group. 

### 4.3. Game-Theoretic Modeling of Gene Interactions

The integration of the part-whole relationship theory and the mathematical aspect of evolutionary game theory allows us to characterize how each gene interacts with every other gene across samples by formulating a system of ordinary differential equations (ODEs). Suppose we want to reconstruct *L*-node coarse-grained networks at the module level. For a particular group of neuroblastoma risk *k* (*k* = for low and 2 for high), such an *L*-dimensional system of ODEs can be written as

(2) $$g_{k}^{\prime }\left(E_{i}\right)=\left[\begin{array}{c} \frac{dg_{1k}\left(E_{i}\right)}{dE_{i}}\\ \vdots \\ \frac{dg_{Lk}\left(E_{i}\right)}{dE_{i}} \end{array}\right]=\left[\begin{array}{c} Q_{1k}(g_{1k}\left(E_{i}):\theta_{1k}\right)+\sum_{j=2}^{L}Q_{1jk}(g_{jk}\left(E_{i}\right):\theta_{1jk})\;\;\\ \vdots \\ Q_{Lk}(g_{Lk}\left(E_{i}):\theta_{Lk}\right)+\sum_{j=1}^{L-1}Q_{Ljk}(g_{jk}\left(E_{i}\right):\theta_{Ljk})\; \end{array}\right]$$

where $g_{1k}\left(E_{i}\right)$ is the expression level of gene *j* on sample *i* (here *i* = 1, …, *n_k_*) from risk group *k*, and the derivative of expression of each gene *j* is partitioned into the *independent* component that occurs if this gene is assumed to be in isolation and *dependent* component that is the aggregated effect of the influence of all other genes on this gene. Function $Q_{jk}(g_{jk}\left(E_{i}):\theta_{jk}\right)$, specifies how gene *j* is expressed independently as a function of EI and is determined by parameters $\theta_{jk}$, whereas function $Q_{jj^{\prime }k}(g_{j^{\prime }k}\left(E_{i}\right):\theta_{jj^{\prime }k})$, describes how gene $j^{\prime }$ affects the expression of gene *j* as a function of EI and is determined by parameters $\theta_{jj^{\prime }k}$ ($j^{\prime }$ = 1, …, *j* − 1, *j* + 1, …, *L*). Unlike classic ODEs with respect to time, ODEs in Equation (1) are specified by the EI derivative, which are thus called quasi-dynamic ODEs (qdODEs) [18].

### 4.4. Variable Selection

Network theory states that there is a cognitive limit to the number of links an individual can stably maintain in a cohesive network [34]. This so-called Dunbar’s law, originally observed in primate societies, can be explained to be due to the limit of the volume of the neocortex. We argue that Dunbar’s law may also be at play in the network constituted by other biological entities, such as cells and genes. The limit to the number of gene–gene interactions exists in living cells, because it is unlikely that each gene interacts with all other genes to form a completely linked network.

To detect a subset of the most significant genes that interact with a focal gene, we implement LASSO-based variable selection. Let $y_{jk}\left(E_{i}\right)$ denote the observed expression level of gene *j* on sample *i* from risk group *k*. A regression model of gene *j* on other genes as predictors, implemented with qdODEs of Equation (2), can be formulated as

(3) $$y_{jk}\left(E_{i}\right)=P_{jk}\left(E_{i}\right)+\overset{L}{\underset{j\prime =1,j\prime \neq j}{\sum }}P_{jj\prime k}\left(E_{i}\right)+e_{jk}\left(E_{i}\right),$$

where the first two terms at the right side characterize the independent and dependent expression components of gene *j* as a function of $E_{i}$ for risk group *k*, and $e_{jk}\left(E_{i}\right)$ is the residual error of gene *j* on sample *i* from risk group *k*, obeying a multivariate normal distribution with mean vector **0** and sample-dependent covariance matrix $\Sigma_{jk}$ for gene *j*. We assume that the residual errors of gene expression are independent among samples so that $\Sigma_{jk}$ is structured as $\Sigma_{jk}\;=\;\sigma_{jk}^{2}I_{n_{k}}$ where $\sigma_{jk}^{2}$ is the residual variance of gene *j* at the same sample and $I_{n_{k}}$ is the identity matrix. 

We use power Equation (1) to model the independent expression component, specified by parameter vector $\theta_{jk}$, and a non-parametric approach to model the dependent expression component, specified by parameter vector $\theta_{jj^{\prime }k}$. We implement group LASSO [35] and adaptive group LASSO [36] to select the most significant genes that link with gene *j* for each risk group. After variable selection, the number of genes that are involved in the dependent component of gene *j* will reduce from *L* to $D_{jk}$, making full qdODEs of Equation (2) become sparse ones. We pose a constraint on the number of regulated genes by a regulator but no constraint on the number of regulators. Through reconstructing high-dimensional but sparse networks by the sparse qdODEs, this gives us a full capacity to identify all possible regulators.

### 4.5. Likelihood and Test

We formulate a likelihood of expression data of *L* modules from two risk groups, as a function of qdODE parameters and residual (co)variances. Assuming that residual errors are independent between the two risk groups, this likelihood is written as

(4) $$L_{1}=\overset{2}{\underset{k=1}{\prod }}f_{k}\left(y_{1k},\ldots ,y_{Lk}|\mu_{1k},\ldots ,\mu_{Lk};\Sigma_{k}\right)$$

where vector $y_{jk}=(y_{jk}\left(E_{1}\right)$, …, $y_{jk}\left(E_{n_{k}}\right))$ denotes the observed expression levels of gene j (j = 1, …, L) on $n_{k}$ samples from risk group k, and $f_{k}\left(\cdot \right)$ is the multivariate normal probability density function with mean vector ($\mu_{1k},\ldots ,\mu_{Lk}$) (with $\mu_{jk}=(\mu_{jk}\left(E_{1}\right)$, …, $\mu_{jk}\left(E_{n_{k}}\right))$) and covariance matrix $\Sigma_{k}$ for risk group k. Here, we assume that microarray gene expression data follow a normal distribution, but other forms of distribution, such as Poisson or negative binomial for RNA-seq expression data, can also be considered. By implementing sparse qdODEs to model the mean vector, we obtain the maximum likelihood estimates (MLEs) of risk-specific ODE parameters $\theta_{jk}$ and $\theta_{jj^{\prime }k}$ (j = 1, …, L; $j^{\prime }$ = 1, …, j − 1, j + 1, …, $D_{jk}$) for each gene for risk group k.

We develop a statistical procedure for testing whether gene networks can explain differences between high and low neuroblastoma risks. Under the assumption of no risk-specific difference, we formulate the likelihood as follows

(5) $$L_{0}=f\left(y_{1},\ldots ,y_{L}|\mu_{1},\ldots ,\mu_{L};\Sigma \right)$$

where vector $y_{j}=\left(y_{j}\left(E_{1}\right),\ldots ,y_{j}\left(E_{n}\right)\right)$ denotes the observed expression levels of gene *j* (*j* = 1, …, *m*) on all *n* mixed samples from two risk groups. Similarly, by implementing sparse qdODEs to model the mean vector, we obtain the MLEs of risk-agnostic ODE parameters $\theta_{j}$ and $\theta_{jj^{\prime }}$ (*j* = 1, …, *L*; $j^{\prime }$ = 1, …, *j* – 1, *j* + 1, …, $D_{j}$) for each gene. Note that $D_{j}$ is the number of regulated genes by gene *j* as a regulator, which is determined through variable selection on a regression model of gene *j* on all other *L* – 1 genes across *n* samples.

By plugging in the MLEs of model parameters into likelihoods (4) and (5), we obtain the likelihood values ${\overset{´}{L}}_{1}$ (assuming that there is a risk-specific difference) and ${\overset{´}{L}}_{0}$ (assuming that there is no risk-specific difference), respectively. We further estimate the log-likelihood ratio

(6) $$\mathrm{LR}=-2\log({\overset{´}{L}}_{0}/{\overset{´}{L}}_{1})$$

as a statistic used to test if *n* samples should be sorted into *C* contexts. By reshuffling *n* samples randomly into two risk groups, we calculate the LR value. If this permutation procedure is repeated 1000 times, we obtain the 95th percentile from 1000 LR values and use it as a critical threshold. 

### 4.6. idopNetwork Recovery

If the risk group is tested to be significantly different from one another, we use the MLEs of $\theta_{jk}$ and $\theta_{jj^{\prime }k}$ to estimate the integrals of $Q_{jk}(g_{jk}\left(E_{i}):\theta_{jk}\right)$ and $Q_{jj^{\prime }k}(g_{j^{\prime }k}\left(E_{i}\right):\theta_{jj^{\prime }k})$, denoted as $P_{jk}(E_{i})$ and $P_{jj^{\prime }k}(E_{i})$ (*j* = 1, …, *L*; $j^{\prime }$ = 1, …, *j* – 1, *j* + 1, …, $D_{jk}$), respectively. We encapsulate $P_{jk}(E_{i})$ as nodes and $P_{jj^{\prime }k}(E_{i})$ as edges into a graph as an *L*-dimensional gene regulatory network G($E_{i}$). This network can capture all three possible features of gene interactions—bidirectional, weighted, and signed—because $P_{jj^{\prime }k}(E_{i})$ can characterize the strength and sign (promotion vs. inhibition) with which gene $j^{\prime }$ affects *j,* and also because $P_{jj^{\prime }k}(E_{i})$ and $P_{j^{\prime }jk}(E_{i})$ can describe and compare how genes *j* and $j^{\prime }$ are reciprocally affected. Relative to most existing networks that do not meet these three features simultaneously, G($E_{i}$) is regarded as being fully informative.

G($E_{i}$) is a function of $E_{i}$, suggesting that we can reconstruct a network for each sample, i.e., patient. To the end, we can reconstruct *n* personalized networks and compare how the networks vary structurally and functionally from one patient to next. If the same patient is transcriptionally monitored at multiple timepoints and/or under multiple treatments, we can reconstruct spatiotemporal network for this specific patient. Increasing evidence shows that a complex disease is controlled by a full set of genome-wide genes [37], indicating the necessity of reconstructing an omnigenic network. Although it is highly challenging to reconstruct large networks, Chen et al. [18] has integrated developmental modularity and Dunbar’s law, which enables them to reconstruct G($E_{i}$) from high- or even ultrahigh-dimensional data of genes. Taken together, we will reconstruct informative, dynamic, omnidirectional, and personalized networks (idopNetworks).

### 4.7. Neuroblastoma Expression Data

We downloaded 23,434-gene expression data of neuroblastoma patients from TARGET website [38]. The data include 247 patients, of whom 217 and 30 belong to high- and low-risk groups, respectively. For each patient, age, gender, MYCN status, PLOIDY, stage, race, risk, overall survival and event free survival are provided. In this study, we focus on the investigation of how immune genes interact with each other to cause risk-specific discrepancies. Although the model can analyze any number of genes, as the proof of concept, we chose 3439 genes that are shown to display various immune-related functions from InnateDB [39]. Using these genes, we reconstructed immune-related idopNetworks for neuroblastoma risk.

### 4.8. Data and Code Availability 

Uploaded at [38,39,40].

## 5. Conclusions

We have developed a computational model to reconstruct and implement fully informative gene networks (idopNetworks) as the biomarkers of neuroblastoma risk. The major advantage of this model lies in its capacity to encapsulate all possible genes into well-organized networks and characterize how gene interaction architecture alters in response to developmental and environmental regimes to regulate biological processes underlying neuroblastoma. The new model may overcome an intrinsic limitation of using individual genes to classify and predict a human disease that virtually includes a number of interactive genes. The technical merit of the new model is to infer context-specific networks from static gene expression, thus greatly facilitating the widespread use of the model to disentangle the complexities of neuroblastoma. As a proof of concept, we used this model to analyze a published transcriptional dataset for 247 patients at high- and low-risk levels of neuroblastoma, from which several important gene modules and interactions were identified to distinguish between these two risk levels. We confirmed the biological relevance of known genes, and also characterized previously unknown gene functions. These unknown genes may potentially provide candidates oncologists use to further investigate the genomic underpinnings of neuroblastoma. 

## Supplementary Materials

The following are available online at https://www.mdpi.com/2072-6694/12/8/2086/s1, Figure S1: Plots of residuals from the fitting of the power equation vs. predicted values for of four randomly chosen genes, *AATK* (A), *AKAP11* (B), *CD8A* (C), and *CDH9* (D), with expression index (EI) for low-risk (red) and high-risk patients (green). The ticks at the *x*-axis indicate the expression indices of samples, Figure S2: Voronoi treemaps that visualize fine-grained idopNetworks among genes from module 2 for high-risk and low-risk patients. Each polygon area (node) is represented by a gene (with its name shown), with the color metric being proportional to the overall expression level of this gene. Activation and inhibition are denoted by arrowed red and blue lines, respectively, with the thickness of lines being proportional to the strength of microbial interactions, Figure S3: Voronoi treemaps that visualize fine-grained idopNetworks among genes from module 38 for high-risk and low-risk patients. Each polygon area (node) is represented by a gene (with its name shown), with the color metric being proportional to the overall expression level of this gene. Activation and inhibition are denoted by arrowed red and blue lines, respectively, with the thickness of lines being proportional to the strength of microbial interactions, Figure S4: Voronoi treemaps that visualize fine-grained idopNetworks among genes from module 31 for high-risk and low-risk patients. Each polygon area (node) is represented by a gene (with its name shown), with the color metric being proportional to the overall expression level of this gene. Activation and inhibition are denoted by arrowed red and blue lines, respectively, with the thickness of lines being proportional to the strength of microbial interactions.
