# Supplementary material for: Computational Identification of Gene Networks as a Biomarker of Neuroblastoma Risk
Source: Cancers (Basel). 2020 Jul 28;12(8):2086. doi: 10.3390/cancers12082086 (PMC7465094; doi:10.3390/cancers12082086)
Supplement: Supplementary file 1 [file cancers-12-02086-s001.pdf]

Supplementary Materials: Computational Identification of Gene Networks as a Biomarker of Neuroblastoma Risk

Lidan Sun, Libo Jiang, Christa N. Grant, Hong-Gang Wang, Claudia Gragnoli, Zhenqiu Liu and Rongling Wu

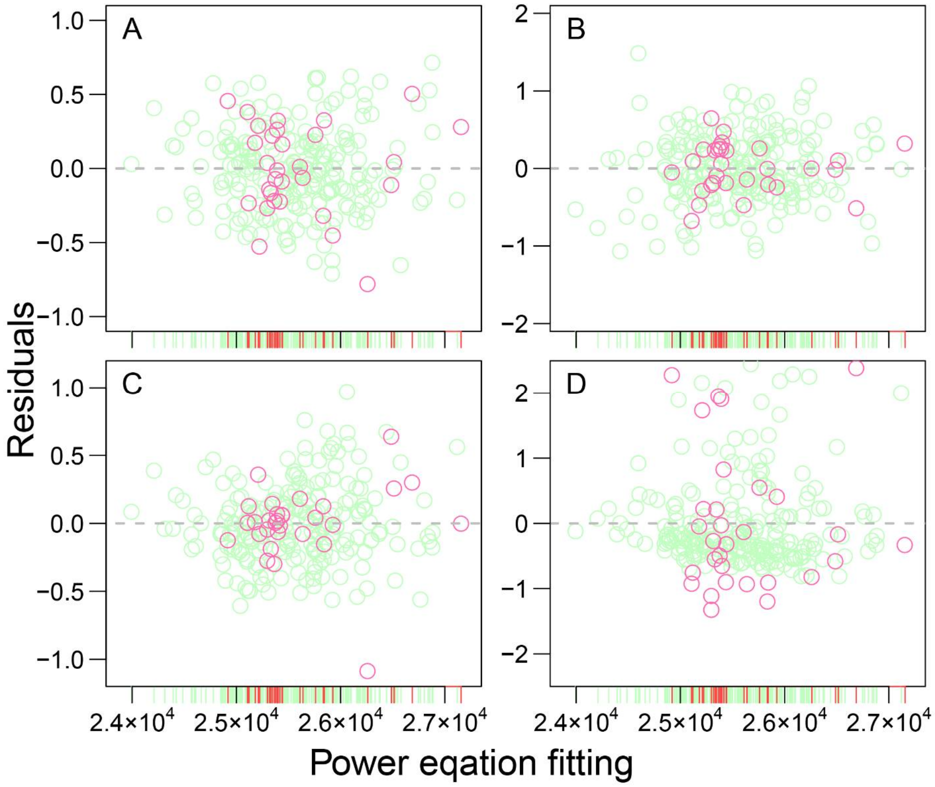

Figure S1. Plots of residuals from the fitting of the power equation vs. predicted values for of four randomly chosen genes, *AATK* (**A**), *AKAP11* (**B**), *CD8A* (**C**), and *CDH9* (**D**), with expression index (EI) for low-risk (red) and high-risk patients (green). The ticks at the *x*-axis indicate the expression indices of samples.

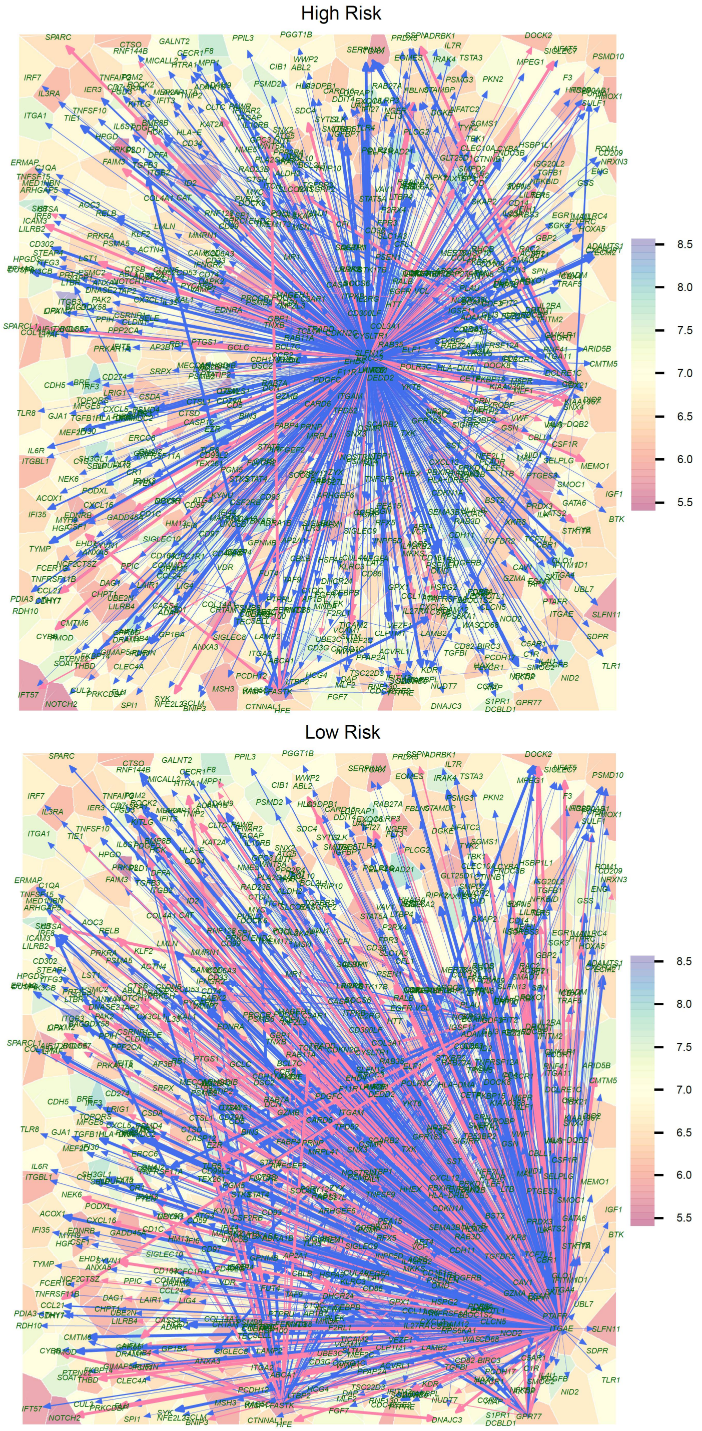

Figure S2. Voronoi treemaps that visualize fine-grained idopNetworks among genes from module 2 for high-risk and low-risk patients. Each polygon area (node) is represented by a gene (with its name shown), with the color metric being proportional to the overall expression level of this gene. Activation and inhibition are denoted by arrowed red and blue lines, respectively, with the thickness of lines being proportional to the strength of microbial interactions.

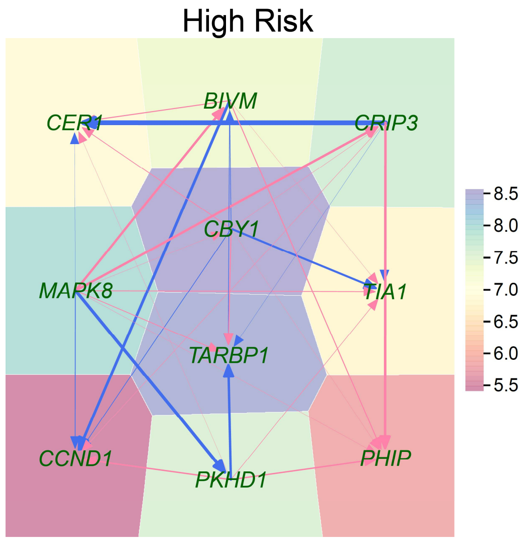

Figure S3. Voronoi treemaps that visualize fine-grained idopNetworks among genes from module 38 for high-risk and low-risk patients. Each polygon area (node) is represented by a gene (with its name shown), with the color metric being proportional to the overall expression level of this gene. Activation and inhibition are denoted by arrowed red and blue lines, respectively, with the thickness of lines being proportional to the strength of microbial interactions.

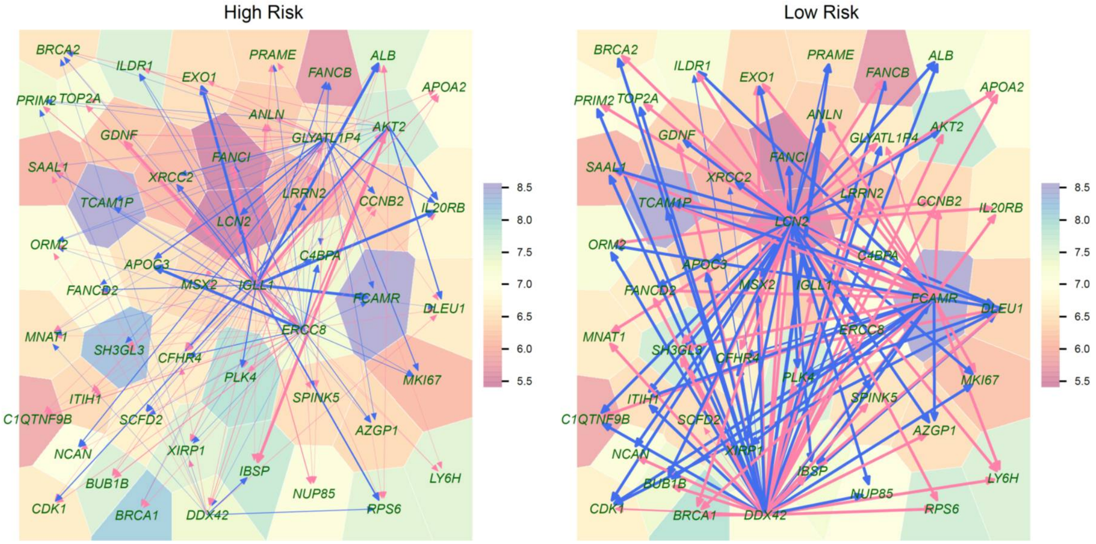

Figure S4. Voronoi treemaps that visualize fine-grained idopNetworks among genes from module 31for high-risk and low-risk patients. Each polygon area (node) is represented by a gene (with its name shown), with the color metric being proportional to the overall expression level of this gene. Activation and inhibition are denoted by arrowed red and blue lines, respectively, with the thickness of lines being proportional to the strength of microbial interactions.

© 2020 by the authors. Licensee MDPI, Basel, Switzerland. This article is an open access article distributed under the terms and conditions of the Creative Commons Attribution (CC BY) license (http://creativecommons.org/licenses/by/4.0/).
